# Supplementary material for: Splice-Junction-Based Mapping of Alternative Isoforms in the Human Proteome
Source: Cell Rep. Author manuscript; Available in PMC 2020 Jan 15. (PMC6961840; doi:10.1016/j.celrep.2019.11.026)
Supplement: 2 [file NIHMS1546469-supplement-2.zip › DF1/PXD010154/Heart/Heart_43_PPP1R12A_ISEMEEELKR.pdf]

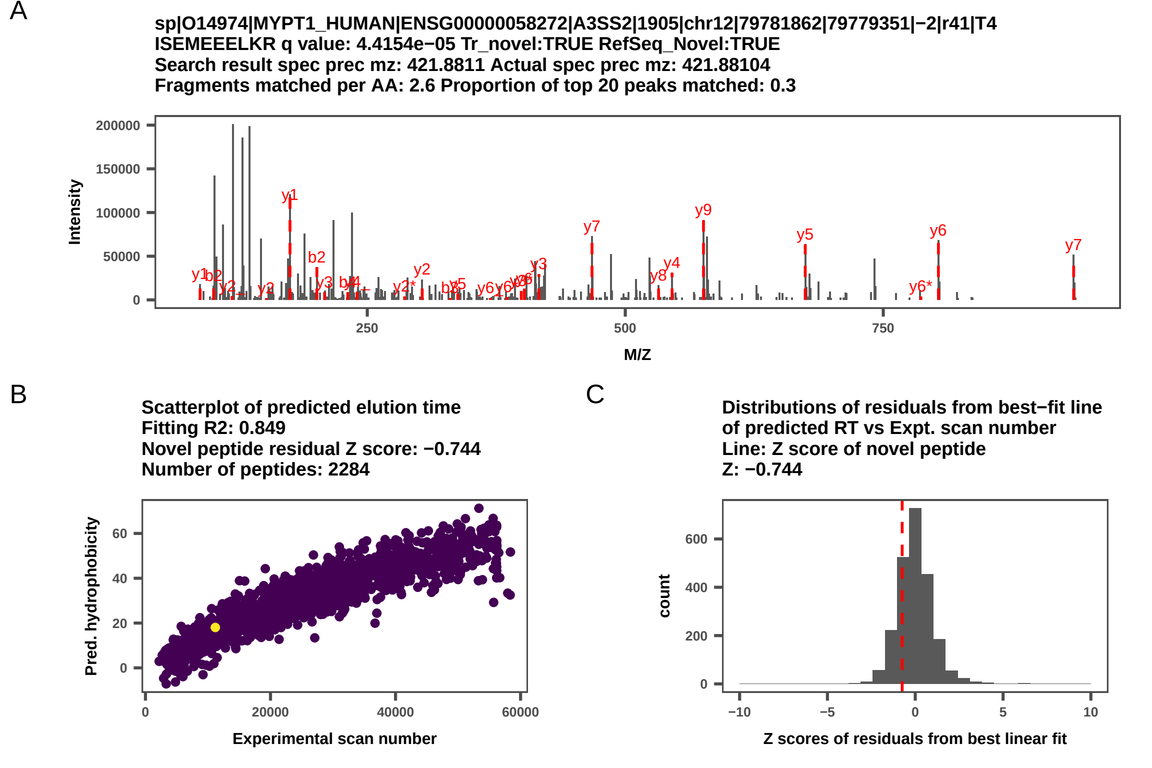
A
sp|O14974|MYPT1_HUMAN|ENSG00000058272|A3SS2|1905|chr12|79781862|79779351|−2|r41|T4
ISEMEEELKR q value: 4.4154e−05 Tr_novel:TRUE RefSeq_Novel:TRUE
Search result spec prec mz: 421.8811 Actual spec prec mz: 421.88104
Fragments matched per AA: 2.6 Proportion of top 20 peaks matched: 0.3
Intensity
200000
150000
100000
50000
0
y1
b2
y3
y2
y2*
y7
y9
y5
y6
y7
y8
y4
y6*
250
500
750
M/Z
B
Scatterplot of predicted elution time
Fitting R2: 0.849
Novel peptide residual Z score: −0.744
Number of peptides: 2284
Pred. hydrophobicity
60
40
20
0
0
20000
40000
60000
Experimental scan number
C
Distributions of residuals from best−fit line
of predicted RT vs Expt. scan number
Line: Z score of novel peptide
Z: −0.744
count
600
400
200
0
−10
−5
0
5
10
Z scores of residuals from best linear fit
